# Performance Comparison of Ambient Ionization Techniques Using a Single Quadrupole Mass Spectrometer for the Analysis of Amino Acids, Drugs, and Explosives

**DOI:** 10.1021/jasms.4c00277

**Published:** 2024-09-02

**Authors:** Simone Mathias, Marius Amerio-Cox, Toni Jackson, David Douce, Bryan McCullough, Ashley Sage, Peter Luke, Carol Crean, Patrick Sears

**Affiliations:** † School of Chemistry and Chemical Engineering, University of Surrey, Guildford GU2 7XH, U.K.; ‡ Waters Corporation, Stamford Avenue, Wilmslow SK9 4AX, U.K.; § Mass Spec Analytical, Future Space UWE North Gate, Bristol BS34 8RB, U.K.

## Abstract

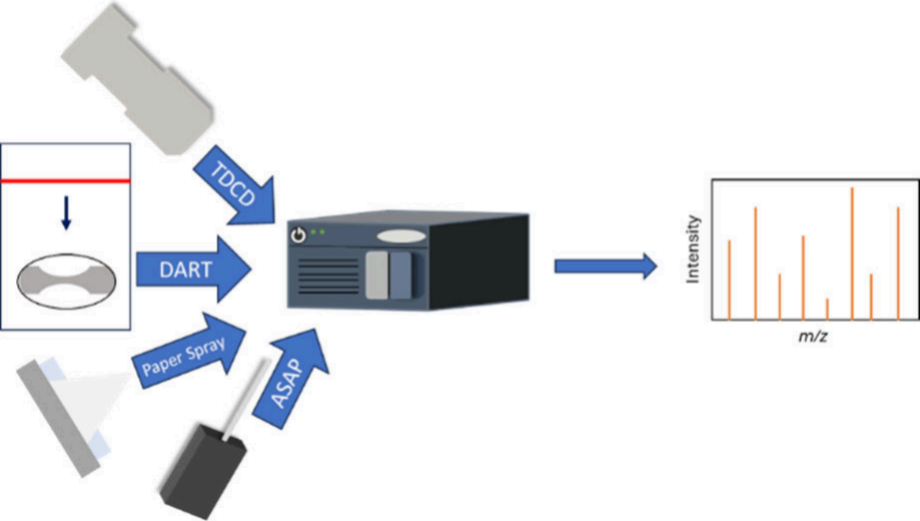

The utilization of ambient ionization (AI) techniques for mass spectrometry (MS) has significantly grown due to their ability to facilitate rapid and direct sample analysis with minimal sample preparation. This study investigates the performance of various AI techniques, including atmospheric solids analysis probe (ASAP), thermal desorption corona discharge (TDCD), direct analysis in real time (DART), and paper spray coupled to a Waters QDa mass spectrometer. The focus is on evaluating the linearity, repeatability, and limit of detection (LOD) of these techniques across a range of analytes, including amino acids, drugs, and explosives. The results show that each AI technique exhibits distinct advantages and limitations. ASAP and DART cover high concentration ranges, which may make them suitable for semiquantitative analysis. TDCD demonstrates exceptional linearity and repeatability for most analytes, while paper spray offers surprising LODs despite its complex setup (between 80 and 400 pg for most analytes). The comparison with electrospray ionization (ESI) as a standard method shows that ambient ionization techniques can achieve competitive LODs for various compounds such as PETN (80 pg ESI vs 100 pg ASAP), TNT (9 pg ESI vs 4 pg ASAP), and RDX (4 pg ESI vs 10 pg ASAP). This study underscores the importance of selecting the appropriate ambient ionization technique based on the specific analytical requirements. This comprehensive evaluation contributes valuable insights into the selection and optimization of AI techniques for diverse analytical applications.

## 1 Introduction

It is widely accepted that for quantitative analysis requiring mass spectrometry data, liquid chromatography–mass spectrometry (LC-MS) and gas chromatography–mass spectrometry (GC-MS) are the gold standard techniques.^1−5^ These techniques allow for high throughput analysis and provide accurate and reliable data.^6,7^ The drawbacks of such instrumentation include initial instrument expense, staff training, and maintenance costs. In addition to these points, the instrumentation is often of a substantial size and weight and analysis can usually only be performed within a laboratory setting.^8^ The development of miniature mass spectrometers has been an area of research since the early 1990s, and significant advancements have been made in this field.^9,10^ Miniature mass spectrometers allow for rapid in situ analysis of samples, which is particularly beneficial when coupled with an ambient ionization sample introduction technique. A review article published in 2016 by Synder et al. shows an extensive list of miniature and deployable mass spectrometers that have been developed both in academic laboratories and by commercial manufacturers.^11^

Ambient ionization (AI), first published in literature in 2004, has been an emerging area of research over the last two decades.^12,13^ Unlike traditional ionization techniques coupled to mass spectrometers (MS), ambient ionization allows for the formation of ions outside of the mass spectrometer without separation and minimal sample preparation, allowing results to be generated at greater throughput than can typically be achieved with LC-MS and GC-MS. When coupled with miniature or deployable mass spectrometers, analysis of samples can take place in situ,^14^ which is beneficial for many application areas such as forensics, environmental, pharmaceutical, health care, security, food and beverages, cosmetics and agriculture.

Many ambient ionization techniques have been developed since the early 2000s and typically exploit well-known ionization processes such as electrospray ionization (ESI) and atmospheric pressure chemical ionization (APCI). Early ambient ionization techniques reported include direct analysis in real time (DART) from Robert Cody’s group and desorption electrospray ionization (DESI) from Graham Cook’s group, both of which are commercially available ion sources.^12,13^ A range of other ambient ionization techniques have been developed and have become commercially available such as atmospheric pressure solids analysis probe (ASAP),^15^ secondary electrospray ionization (SESI),^16^ probe electrospray ionization (PESI),^17^ paper spray ionization (PS),^18^ and liquid extraction surface analysis (LESA).^19^ In addition to the techniques listed above, many others have been developed in laboratories for specific applications and are often “homemade” designs. For example, thermal desorption corona discharge (TDCD),^20^ low temperature plasma,^21^ dielectric barrier discharge ionization,^22^ laser ablation electrospray ionization,^23^ and touch spray to name a few.^24^

**Figure 1:**
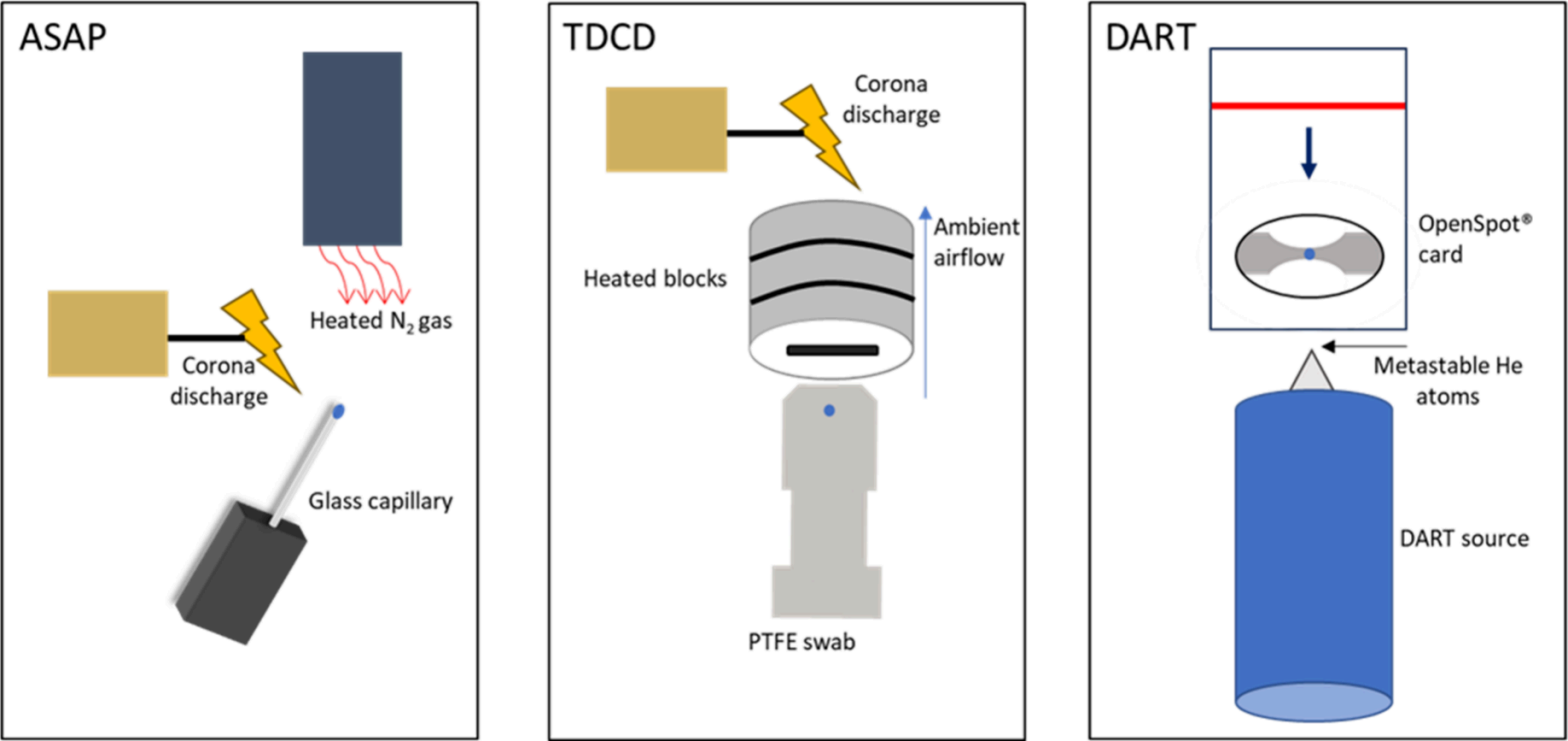
Schematics depicting the sample introduction and ionization type for ASAP, TDCD, and DART.

Figure 1 depicts the experimental setup of ASAP, TDCD, and DART and the sample introduction techniques used within this study. DART uses gas, normally helium or nitrogen, that passes into a chamber where a needle with a high electrical potential creates an electrical discharge. This produces excited state species, electrons, and ions within a plasma which passes through an electrode which can be biased to remove unwanted charged species, allowing metastable atoms to enter the ionization region. As the gas exits the ion source, it contains only electronically excited, neutral species which can then interact with a sample directly, releasing gas-phase ions before reaching the inlet of the mass spectrometer.^12^

ASAP and TDCD discharges are similar. ASAP utilizes a hot gas stream, typically nitrogen, to desorb an analyte from a borosilicate glass melting point tube. The gas-phase molecules are then ionized by a corona discharge before reaching the inlet of the mass spectrometer. Further details can be found in our previous publication using ASAP for the detection of explosives.^25^ In the case of thermal desorption corona discharge mass spectrometry, a sample, usually on a swab or similar material, is inserted between heated blocks which thermally desorb and volatilize target analytes from the surface. Gas-phase molecules travel through a ceramic transfer line before being ionized by a corona discharge needle, and the resulting ions then travel toward the MS inlet for detection.^20,26^

Paper spray allows for direct analysis of samples by mass spectrometry by placing a sample on a piece of paper with a sharp point. A spray solvent is used to transport the sample to the tip of the paper, while a high voltage is applied to perform ionization by producing charge droplets through a Taylor cone. Research has been conducted by Bailey et al. using paper spray for the detection of explosives and antipsychotic drugs.^27,28^ Paper spray is similar to ESI and does not require a vaporization step, but the analyte must be extracted from the paper by the spray solvent.

AI techniques are versatile in terms of which instruments they can be coupled to, which has allowed for a wide range of applications to be explored. While this is beneficial in terms of the amount of literature available, it does not provide much information on which ambient ionization technique may be best suited for a particular type of analysis, as performance will vary between design and mass spectrometer used. The British Mass Spectrometry Society (BMSS) conducted two ambient ionization interlaboratory studies in 2015 and 2017. Study 1 focused on the detection and quantitation capabilities using a wide range of analytes to include different chemistries, while study 2 provided the same eight-component pharmaceutical drug sample to participants for analysis using their choice of ambient ionization technique and mass spectrometer, each with their own experimental conditions in order to investigate the robustness and repeatability of the techniques.^29^ A key part of the conclusions of this study was that it was not possible to draw firm conclusions from the data set, likely due to the diversities in experimental setup. Additionally, it was found that some of the spray techniques struggled to detect the lower-mass analytes, potentially due to the increased chemical noise in this mass region, while the corona discharge and plasma-based techniques did not seem to have issues in this respect. A limitation of the BMSS studies was that a range of different mass spectrometers has been used from high resolution orbitraps and QToFs to ToFs and quadrupoles (both triple and single), which impacts the ability to detect analytes within a sample, not to mention the differences between manufacturer instruments’ ionization processes. Additionally, there will be variability in the performance of prototype and homemade ambient ionization sources compared to those that are commercially available products. We have previously shown that the ionization source can impact the selectivity and sensitivity of the ions produced for a number of explosive compounds.^30^

The aim of this study was to carry out an objective comparison of ambient ionization techniques using the same mass spectrometer, which resulted in the use of prototype sources, as commercially available sources are often instrument/manufacturer specific. The work presented in this paper utilizes a range of different ambient ionization techniques including ASAP, TDCD, DART, and PS coupled to a Waters Acquity QDa mass spectrometer to assess other analytical parameters, which would be required for a quantitative application.^31−33^ The parameters investigated include limit of detection (as a measure of sensitivity), linearity, and repeatability for a set of analytes. The analytes were chosen to represent routinely screened materials and belong to the three categories: illicit drugs, amino acids, and explosives.

## 2 Experimental Section

### 2.1 Materials

Optima LC-MS grade methanol, acetonitrile, 2-propanol, formic acid, and water were purchased from Fisher Scientific (Leicestershire, UK). Leucine and phenylalanine were obtained from LGC (Middlesex, UK). Amphetamine, ketamine, (−)-*trans*-Δ^9^-tetrahydrocannabinol (THC), and cocaine were purchased from Ceriliant, Sigma-Aldrich (Dorset, UK). PETN, RDX, TNT, Tetryl, and HMTD were purchased from AccuStandard, Cole-Parmer (Cambridgeshire, UK). Ammonium chloride, ammonium nitrate, ammonium formate, ammonium acetate, and lithium acetate were purchased from Sigma-Aldrich (Dorset, UK).

Borosilicate glass melting point tubes were purchased from Fisher Scientific (Leicestershire, UK) for use with ASAP. Itemizer sample traps (Teflon-coated fiberglass swabs) were purchased from DSA Detection (Hertfordshire, UK) for use with the thermal desorber corona discharge. Whatman 1 chromatography paper 200 × 200 mm was purchased from Sigma-Aldrich (Dorset, UK) and cut into paper triangles (1.61 × 2.1 cm, base × height) as previously described by Costa et al. for use with paper spray.^34^ OpenSpot cards were obtained from IonSense for use with DART.

### 2.2 Sample and Standard Preparation

#### 2.2.1 Direct Injection by Electrospray Ionization

Phenylalanine and leucine were dissolved in water before being diluted in 50:50 methanol and 0.1% formic acid. Amphetamine, ketamine, THC, and cocaine were diluted in 50:50 methanol–water and 0.1% formic acid. Tetryl, TNT, RDX, and PETN were prepared in 50:50 methanol–water with 1 mM ammonium nitrate, ammonium chloride, ammonium acetate, or ammonium formate. HMTD was prepared in the same manner but with 1 mM lithium acetate. Dilute samples were prepared volumetrically from stock solutions covering the concentration range 0.001 μg/mL to 0.4 μg/mL.

#### 2.2.2 Ambient Ionization

Phenylalanine and leucine were dissolved in water before dilution in methanol. All other standards were diluted in methanol covering the following concentration range of 0.25–100 μg/mL.

### 2.3 Experimental Setup

All analyses were performed on the Waters QDa mass spectrometer (Waters, Wilmslow, UK) using ESI, ASAP, DART, TDCD, and paper spray, which will be detailed further below. The system was operated in performance mode with an external rotary pump (Vacuubrand RE 6) and 0.2 mm aperture. The optimized conditions (based on the largest signal intensity for the most abundant ion) for each analyte were investigated by using each technique to improve sensitivity.

Initial experiments were conducted using the ESI source (Figure S1 in the Supporting Information) coupled to a Waters Acquity LC to allow for direct injection of the sample without a column present. A 50:50 MeOH:water solution was used to carry the sample to the MS with a flow rate of 0.5 mL/min with an injection volume of 2 μL.

For experiments carried out with the prototype thermal desorption corona discharge (TDCD) source (MSA, Bristol, UK), an external power supply (Stanford Research Systems, Sunnyvale, CA) was used to provide a discharge voltage to the corona pin, and the temperature of the heated elements of the source was controlled by a Rapid Analysis Controller (MSA, Bristol, UK). The temperature was set to 225 °C for analysis of leucine, phenylalanine, amphetamine, ketamine, THC, and cocaine, 275 °C for TNT, Tetryl, PETN, and RDX, and 150 °C for HMTD. The corona pin was set to +/–3 kV depending on ionization mode. An external vacuum pump was used to pull gas-phase species toward the inlet of the MS (Dwyer Instruments, Michigan City, IN) at approximately 20 L/min. A schematic of the TDCD can be seen in Figure S2 of the Supporting Information.

A prototype ASAP source (Waters, Wilmslow, UK) was coupled to the QDa, and the external power supply (as above) was used to provide the voltage for the corona pin (set to ±3 kV depending on ionization mode). The probe temperature, which heats the nitrogen gas (flow of approximately 2 L/min), was set to 450 °C for analysis of all compounds, except HMTD where it was set to 200 °C. Figure S3 in the Supporting Information shows a schematic of the ASAP source.

An in-house designed paper spray source (Figure S4 in the Supporting Information) was connected to the external power supply (as above) to provide high voltage (+3 kV for positive ionization (for HMTD, +1.75 kV was used) and −2 kV for negative ionization) to the solvent deposited on the paper triangles. For the analysis of the drugs and amino acids, a spray solvent of acetonitrile and 0.1% formic acid was used. For HMTD, isopropyl alcohol with 0.1% formic acid was used. Analysis of negative-mode explosives used methanol with 0.1 mM ammonium chloride and ammonium nitrate as the spray solvent. In all cases, 30 μL of spray solvent was deposited on the paper triangle. The paper spray source was directed so it was facing toward the inlet of the MS, with the tip of the paper triangle sitting approximately 0.5 cm away from the cone.

A DART source (IonSense, Saugus, MA) was mounted to the QDa using the source housing with a Vapur interface (an additional pump which helps prevent excessive gas flow reaching the mass analyzer). Helium was used as the discharge gas while nitrogen was used when the source was in standby. The gas was heated to 150 °C for the analysis of HMTD and 200 °C for all other analytes. OpenSpot cards were used to introduce samples into the gas stream via the DART OpenSpot source, which was positioned at 1.4 cm on the QDa mount. A schematic of the DART can be found in Figure S5 in the Supporting Information.

To identify analyte ions, the MS was operated in full scan over the *m*/*z* range of 30–500 with varying cone voltages and a scan time of 0.15 s. The source temperature was set to 150 °C. The cone voltage was varied to produce “in-source” fragmentation which allowed for the development of single ion monitoring (SIM) methods to improve sensitivity. The SIM methods for each technique can be found in Table S1 in the Supporting Information with corresponding ions for each compound.

In all cases, the peak area from an extracted ion chromatogram (based on the most abundant ion) was used for data analysis. A total of five replicates per sample was acquired, enabling the repeatability of the techniques to be assessed.

## 3 Results and Discussion

Several analytical parameters have been investigated using ambient ionization techniques, which have been compared to ESI, a routinely used atmospheric pressure ionization source. There is literature demonstrating quantitative applications of AI-MS, but due to the variability in results it is more likely to be adopted for screening purposes or semiquantitative applications. While the linearity of a calibration curve is not essential for a screening application, it is still a good indicator (along with repeatability) of the confidence associated with the results produced by a technique. The analytes used throughout this study were chosen with screening applications in mind: illicit drugs for screening for drugs of abuse, explosives for security aspects, and amino acids for clinical applications.

The following section discusses the results from each technique in the context of LOD, repeatability, and linearity for the amino acids, drugs, and explosive analytes. The LOD was used as an estimate of the sensitivity, the repeatability was established based on the precision of the measurement and was determined by the relative standard deviation, and the linearity was determined on the basis that the coefficient of determination (*R*^2^) was above 0.9 and therefore exhibits linear features.

### 3.1 Paper Spray

All the analytes were tested using paper spray, and this technique was found to be sensitive for analyte detection (see Table S2 in the Supporting Information). However, the results produced were found to be unreproducible in terms of signal response. This was due to a combination of factors including the design of the source, which required alignment with the MS inlet between each analysis. The analyte response was highly variable with sprays lasting between 15 s and 1 min, and this resulted in nonrepeatable peak area responses for the same quantity of analyte. The inconsistency in acquisition spray times may occur due to the paper “drooping” once the spray solvent is applied, resulting in the tip of the paper no longer being aligned with the MS inlet. Research has shown that multiple jet sprays can be produced as the high voltage is applied to the spray solvent deposited on the paper, and while it may increase ionization efficiency, the number and occurrence of these jets may differ per replicate analysis.^35,36^ An increased efficiency of ionization of the analytes through the spray mechanism, particularly if multiple jets form resulting in a larger quantity of analyte reaching the detector even at low concentration, may be the reason for the sensitivity observed; however, this reduces the repeatability.^35,36^

Repeatability was investigated during the early method development stages, where it was common to observe RSDs greater than 50% (*n* = 5). This is unsurprising considering the manual setup of the in-house designed source, resulting in estimating the geometry of the tip of the spray vs the inlet of the mass spectrometer. In addition, paper triangles are cut out with a pair of scissors, which undoubtedly results in minute variations in the angle of the tip which could lead to the spray also being misaligned with the inlet of the mass spectrometer.

Paper spray is time-consuming due to having to prepare the paper triangles, align the source with the instrument inlet, deposit the spray solvent, and apply the high voltage, all of which could result in a failed spray; therefore, it was decided that it would not be used for the assessment of linearity. In this instance, paper spray has been used as a proof of principle of the ionization process, and subsequent variants on the source design could improve on some of the limitations experienced.

### 3.2 Limit of Detection

Estimated limits of detection were established using the ESI source for QDa and for AI techniques. Table 1 reflects the estimated limits of detection and lower limits of quantification (LLOQ). The LOD was calculated by using the blank response produced for the most abundant ion per analyte. Details of the calculation used for determining the LOD can be found in the Supporting Information. The LLOQ reflects the lowest amount of analyte detected within the linear mass ranges described in section 3.3 and Table S3 in the Supporting Information.

**Table 1:** Estimated Limits of Detection and Lower Limits of Quantification Established Using the ESI Source for the QDa and Ambient Ionization Techniques: Atmospheric Solids Analysis Probe, Thermal Desorption Corona Discharge, and Direct Analysis in Real Time

	ASAP		TDCD		DART		ESI	
analyte	LOD (pg)	LLOQ (pg)	LOD (pg)	LLOQ (pg)	LOD (pg)	LLOQ (pg)	LOD (pg)	LLOQ (pg)
amphetamine	130	250	2000	5000	100	500	8	8
ketamine	70	250	500	1000	30	750	6	6
cocaine	30	250	200	250	70	250	1	1
THC	30	250	2000	2000	300	500	80	80
leucine	900	5000	2000	2000	200	1000	60	60
phenylalanine	2500	10000	10000	10000	3000	5000	20	20
HMTD	400	500	1500	5000	1000	1500	60	60
PETN	100	1500	1500	5000	500	500	80	3500
Tetryl	40	250	900	5000	30	250	1.5	2
TNT	4	250	700	1000	100	250	9	10
RDX	10	500	5000	5000	10	1500	4	4

The best limits of detection are observed with the ESI source, which was expected since the technique has a high efficiency for transferring ions from the sample (already in solution) to the detector compared to the ambient ionization techniques. Experiments were carried out using the ESI source to understand the performance of the Waters QDa mass spectrometer and establish a “best case” scenario for the LODs to which the AI techniques could be compared to. The LODs observed with the ambient ionization techniques are higher due to the lower efficiency of the transfer of material from the sample to the detector. In the case of ASAP, TDCD, and DART the sample is solid, as the solvent was left to evaporate before being introduced to the source. This means that the sample must first be vaporized and ionized before reaching the front of the MS, a process that may not be efficient and could result in much of the analyte not reaching the detector. This is particularly evident when using prototype techniques (specifically ASAP and TDCD) where several source variables have not been optimized.

The results shown in Table 2 show that ASAP and DART are the best performing AI techniques and are highly comparable (in most cases) in their analytical performance. While these results may not highlight an overall “top performer” in terms of an AI technique, insight into how compounds may behave within a particular ionization source may be established. For example, the estimated LOD of TNT is strikingly low for ASAP compared with the other AI techniques explored and additionally is very close to the LOD presented for ESI. This suggests that this particular ion source is very sensitive for the analysis of nitroaromatic explosives.

**Table 2:** Relative Standard Deviation (%) at the Middle Point of the Linear Range for Each Analyte and Technique Used

	RSD (%)			
analyte	ASAP	TDCD	DART	ESI
amphetamine	41	11	30	0.4
ketamine	30	31	31	0.7
cocaine	35	26	26	0.7
THC	15	16	30	1.4
leucine	46	18	11	1.0
phenylalanine	45	5	16	0.3
HMTD	23	6	13	2.1
PETN	24	14	27	10
Tetryl	25	12	18	0.3
TNT	14	6	49	1.8
RDX	45	8	24	2.3

Another trend that can be picked out is that TDCD tends to produce higher LODs compared to the other techniques for most analytes. It is likely that this is a product of the higher background noise due to the amount of air being pulled through the hot blocks via the external pump and also a result of the peak associated with a blank swab used in the calculation of the estimated LOD.

### 3.3 Linearity

The mass ranges described in Experimental Section were tested for each ionization technique, and calibration curves were produced to establish what linearity could be achieved. The mass ranges selected were between 0.25–100 ng for the AI techniques and 0.001–0.4 ng for ESI and were chosen as they cover several orders of magnitude. The criteria used to determine if linear features were exhibited was a coefficient of determination (*R*^2^) value >0.9. Ambient ionization is best suited for screening applications where identification is more important than quantification. Sensitivity is a key feature for understanding pass/fail criteria, and repeatability/linearity is more important closer to the LOD to ensure confidence in the result. For these reasons, the maximum amount of material tested for ambient ionization techniques was 100 ng.

The results presented in this section refer to the amount of solid material present as opposed to a concentration, as in the cases of the ambient ionization techniques, the solvent was left to evaporate, leaving behind a solid residue on the sample introduction media. In all cases, the peak area for the most abundant ion for each analyte was used, and five replicates were carried out.

**Figure 2:**
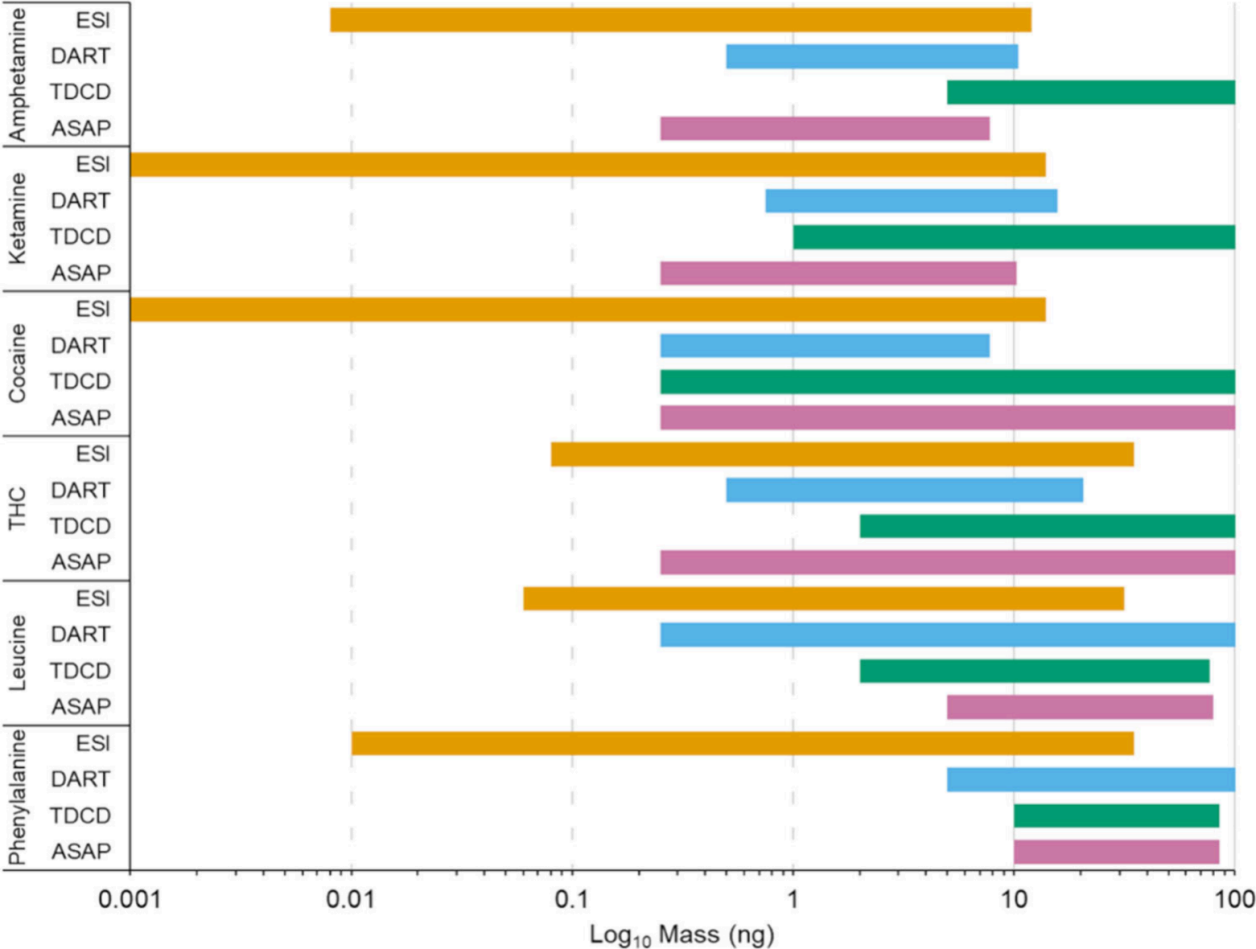
Masses (ng) at which an approximate linear relationship (*R*^2^ > 0.9) was observed for the illicit drugs and amino acids using the ionization techniques ESI, DART, TDCD, and ASAP displayed on a logarithmic scale.

**Figure 3:**
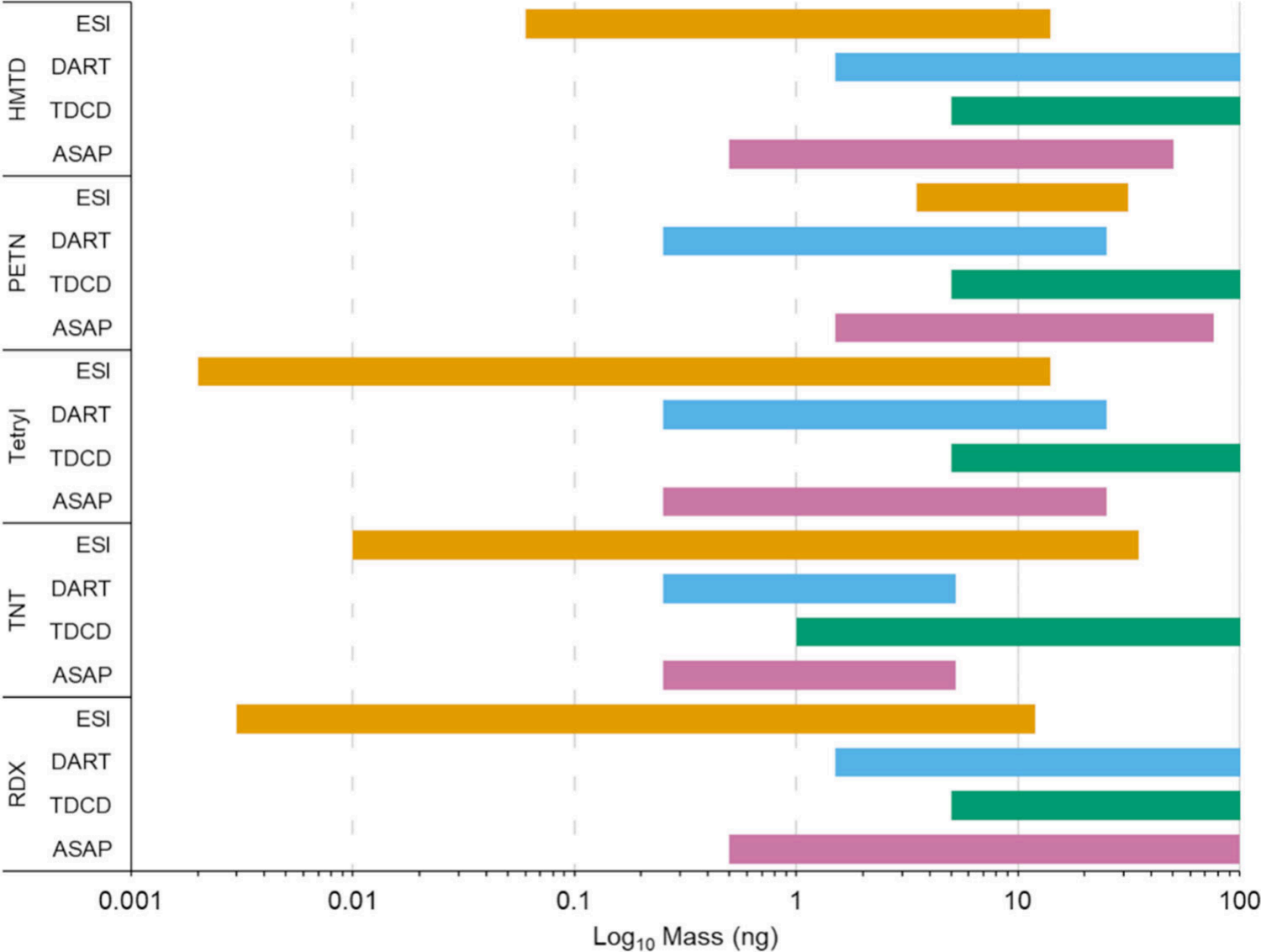
Masses (ng) at which an approximate linear relationship (*R*^2^ > 0.9) was observed for the explosives using the ionization techniques ESI, DART, TDCD, and ASAP displayed on a logarithmic scale.

Figures 2 and 3 depict the mass ranges (on a logarithmic scale) where a linear response was observed for each of the techniques and analytes investigated. It is evident that the results produced by ESI cover the largest concentration range (typically three to 4 orders of magnitude), which shows the achievable dynamic range of the instrument under “ideal conditions”. The results produced by the AI techniques cover smaller ranges of linearity due to reduced ionization efficiencies compared to ESI.

Results of the approximate linear response and corresponding *R*^2^ values produced using ASAP, TD, DART, and ESI for the target analytes can be found in Table S3 in the Supporting Information.

Typically, AI has not been used for quantitative purposes due to the variability associated with the manual sample introduction and the efficiency of desorption/ionization; however, an internal standard can improve such variance. This has been previously shown by a number of researchers, including the BMSS interlaboratory study where the *R*^2^ value of paracetamol increased from 0.9973 to 0.9997 with an isotopically labeled internal standard.^29^ The results produced in this study were fair surprisingly well, considering that no internal standard was present, for these ambient ionization techniques. TD performed exceptionally well, with *R*^2^ values close to 1, with cocaine, THC, HMTD, PETN, tetryl, and TNT having an *R*^2^ value of >0.99.

The ranges presented in Table S3 (Supporting Information) show that a large concentration range can be covered by the majority of these ambient ionization techniques. This may be beneficial in settings where minimal sample preparation is advantageous such as locations where in situ analysis would be desirable (festivals, borders and security settings, clinical scenarios, etc.) and where the amount of analyte may be unknown.^37−40^ In settings where only an indication of a target analyte’s presence is required, it is more important that the linear range is close to the LOD, as this is the point at which a pass or fail decision would be made. The results indicate that ASAP and DART would both be better suited than TDCD, for these types of applications where trace detection is required, as they have better LODs and a linear range that covers lower concentrations.

TDCD offers the capability for semiquantitative aspects at a higher concentration range for most analytes. The results produced by ASAP and DART have similar working ranges, but for amphetamine, ketamine, and TNT, ASAP is capable of covering a lower concentration range compared to the rest of the analytes tested. For TNT, it is likely that the working range is lower than some of the other target analytes due to the estimated limit of detection (as discussed in section 3.2) being 2 orders of magnitude lower than the smallest concentration on the calibration curve. In addition, for several of the analytes tested by ASAP, the concentration at 100 ng was beyond the working range. It should be noted that for the techniques where 100 ng is given as the top concentration, it is possible that the linear relationship may continue past this point.

As mentioned above, the results produced by ESI typically cover a few orders of magnitude but at a much lower concentration compared to the AI techniques, as the relationship between the peak area and these higher concentrations became nonlinear. At these higher concentrations, the detection system became saturated, which suggests that the efficiency of ionization by ESI is much greater than the ambient ionization techniques used in this study.

### 3.4 Repeatability

The inherent variability between replicate analyses can result in large relative standard deviations (RSD). The inclusion of an isotopically labeled internal standard will correct for some of the factors which result in the variance observed in results, such as desorption efficiency, placement of analyte on sampling materials (both location and repeatability of amount deposited each time), and sample introduction to the mass spectrometer. The full precision data for each analyte per technique can be found in the Supporting Information (Table S4).

The Food and Drug Administration (FDA) have published guidelines for bioanalytical method development, and ±15% is considered acceptable for precision when testing nominal concentrations using chromatographic techniques.^41^ The data collected using the ESI source typically produced RSDs below 1% (*n* = 5), which suggests that the use of automated sample introduction offers high levels of precision, due to the well-controlled environment (geometry, gas flows, and temperatures) which are designed for use with liquid chromatography. It is not uncommon for RSDs to be around 50% (*n* = ≥5) for AI techniques when the peak response alone is used, as demonstrated by the BMSS study.^29^ Results produced by the TDCD technique appear to produce the second lowest RSDs with a large number being less than 20% (*n* = 5). In the case of the RSDs produced by ASAP and DART, there was no clear discernible trend whereby one could be recommended for better levels of precision over the other, as in many instances the data are similar, even over differing concentration ranges between the analytes tested. Table 2 reflects the RSD at the middle point of each linear range for each analyte and technique used within this study and shows an example of the variability observed using ASAP and DART. In addition, most of the values shown for the TDCD would fall within the acceptable precision limits set out by the FDA.

For both TDCD and DART, the sample was deposited onto the sample media (fiberglass swabs and OpenSpot cards), and the solvent was left to evaporate, leaving solid residue behind before being introduced into the ionization source. In the case of the TDCD, the fiberglass swab is inserted horizontally into the heated region where the sample is desorbed from the swab surface, and resulting gas-phase molecules are pulled into the ionization region prior to sampling into the mass spectrometer. The OpenSpot cards are placed vertically into the card holder. Here the heated helium gas-phase metastable ions desorb and ionize the analyte which are pulled toward the vacuum region through the Vapur interface by an external vacuum pump. It was expected that the variability would be similar between the two techniques; however, as noted above, the TDCD had better levels of precision. The DART may suffer from greater variability in replicate analyses as the plasma is targeted at a specific point on the OpenSpot card, and it is possible that upon sample deposition the analyte spreads, resulting in different amounts being within the narrow plasma beam each time, compared to the oven-like heated region in the TDCD which results in the whole sample being desorbed each time.

A single measurement is unlikely to be relied upon within any analytical testing environment; however, the rapid nature of analysis when using AI allows for a larger number of replicate analyses to be carried out in a short space of time. In this instance, only five replicates were carried out per concentration and analyte, but depending on the nature of the analysis, a greater number could be more suited and potentially lend itself to improvements in the accuracy with a better estimate of the mean. The main driver behind AI research is based on targeted analysis, where detection is more important, not necessarily a quantitative response.

### 3.5 Advantages and Limitations

Ambient ionization techniques, as described in Introduction, offer the benefit of rapid, direct analysis with minimal sample preparation. A key area where ambient ionization is likely to be of use surrounds applications where a semiquantitative targeted result is desirable, particularly for on-site analysis before confirmation testing can be completed on a complementary instrument in a laboratory environment. The simple nature of most ambient ionization experimental setups, especially when coupled to a small portable mass spectrometer, also lends itself to screening applications. This is particularly relevant where high-throughput analysis is required, such as at airports, border control, and security testing at festivals and other large events. The use of swabs with the TDCD ion source is designed for this type of application and is the most deployable of the ambient ionization techniques described in this work, whereby surfaces, belongings, or people can be sampled using the swab and then directly analyzed afterward. In many of these applications, the amount of sample is unlikely to be a limiting factor, and therefore the higher LODs experienced by the TDCD should not present an issue for detection.

ASAP has reasonable LODs (typically between 100–1000 pg) but can lack repeatability (RSDs on average ∼20–60% at *n* = 5); however, it is easy and simple to use and lends itself well to a targeted workflow. The use of glass rods means that both solid and liquid samples can be easily analyzed, providing that enough sample is available, and is another technique that would work well in the field.

The ASAP source used within this study was a prototype of the commercially available Waters RADIAN system. This prototype had already been optimized in terms of the geometry relating to where the sample meets the clean nitrogen gas, which pushes the ions toward the MS inlet after ionization. In contrast, the TDCD is an early prototype, which uses an external vacuum pump to pull ions (and ambient air) toward the MS inlet, resulting in a higher background signal and lower sensitivity compared to the ASAP. The TDCD offers a lot of potential due to the higher precision (RSD %) but lacks sensitivity compared to the other techniques explored and requires further developmental work to improve on LODs.

In comparison, the DART is quite a bulky setup and requires helium and nitrogen gases, which makes it unsuitable for in-field analysis, despite the simple OpenSpot card sampling; therefore, it is better suited for use within a laboratory setting.

Table 3 reflects the cost per sample and the total time taken for analysis of a sample, including setup of the sampler by the various ambient ionization techniques used in this study. The cost of helium for use with the DART and solvents used for paper spray were found to be negligible and have not been included in calculation per sample. It is evident that the cost of sample analysis by DART is the most expensive, with paper spray being the most time-consuming to carry out.

**Table 3:** Cost of Analysis and Time Taken Per Sample for Analysis for ASAP, TDCD, DART, and Paper Spray

	technique			
	ASAP	TDCD	DART	paper spray
cost of sampler	£0.05 per rod	£0.30 per swab	£2.00 per OpenSpot card	£0.04 per triangle
time per sample	∼30 s	∼30 s	∼30 s	∼3 min
other aspects	requires clean N_2_	requires external air pump	He/N_2_ required, OpenSpot interface	requires solvent, glass slides, and aluminum foil

Since there is no chromatographic separation step when using ambient ionization techniques, the ions will be detected within a single scan or over a small number of scans, which can result in complex spectra being produced. This can be mitigated if targeted analyses are being conducted by using single ion monitoring or multiple reaction monitoring (MRM) methods, where only preselected ions are allowed to reach the detector. In addition, if analyzing a mixture, analytes may compete for ionization, which could result in ion suppression. Isobaric compounds may also present as an issue, especially in instances where identifying fragments cannot be produced using cone voltage (“in-source” fragmentation) or collision energy. The use of tandem MS instruments with an MRM could help to separate isobaric compounds through the production of unique fragments. High resolution mass spectrometry techniques would be required for the separation of target analytes that have a similar mass; however, these instruments are typically only suited for analyses carried out within the laboratory and are more costly compared small footprint mass spectrometers.

## 4 Conclusion

The choice of ambient ionization technique can affect the linearity, repeatability, and limit of detection of measurements for a variety of analytes. It could be beneficial for analysts and researchers to consider which technique is best suited to the analyte of interest when preparing experiments, depending on the desired outcome. For example, the TDCD offers simple sample introduction which results in repeatable measurements (RSDs typically between 10–20%) (for an ambient ionization technique) with good levels of linearity at higher concentrations (*R*^2^ values of >0.99 for around half of the target analytes tested) but may not be best suited for trace detection where sensitivity is important due to the increased background noise. Alternatively, paper spray provides surprisingly low limits of detection, as low as 80 pg for the detection of cocaine, but poor repeatability (RSDs observed are typically ∼60%) and a more complex setup which is unlikely to be of use within routine analysis, especially without fixed geometries and premade paper triangles. Verispray is a commercially available product for Thermo instruments which may answer many of the observed issues discussed within this paper.

The results presented in this study show reasonable levels of precision (typically between 20% and 50%) for the AI techniques considering that no internal standard was used. The spread of results lends itself to poorer accuracy, which would result in calculated concentrations being over- or underestimated when using AI, but this is to be expected with the higher levels of variability when carrying out replicate measurements. The sample is unlikely to be a limiting factor in situations where AI-MS is most likely to be used, particularly where large quantities of sample are present for targeted analysis, e.g., drugs of abuse screening at borders.

Most of the AI techniques used within this study are prototypes or have been designed and developed in house; therefore, it is possible that changes could be made to the ion source to overcome some of the limitations, such as the increased background noise seen on TDCD.

## Data Availability

The data which supports the findings of this study are openly available at Open Science Framework DOI: 10.17605/OSF.IO/WHNQ5

## Supporting Information Available

Additional experimental details and figures of experimental setup of ESI, TDCD, ASAP, paper spray, and DART. Tables describing estimated LOD for paper spray, approximate linear ranges, and full precision data for AI-MS techniques used (PDF)
