# Supplementary material for: Performance Comparison of Ambient Ionization Techniques Using a Single Quadrupole Mass Spectrometer for the Analysis of Amino Acids, Drugs, and Explosives
Source: J Am Soc Mass Spectrom. 2024 Sep 2;35(10):2480–9. doi: 10.1021/jasms.4c00277 (PMC11457451; doi:10.1021/jasms.4c00277)
Supplement: Supplementary file 1 — js4c00277_si_001.pdf [file js4c00277_si_001.pdf]

**Supporting information for**

# Performance Comparison of Ambient Ionization Techniques Using a Single Quadrupole Mass Spectrometer for the Analysis of Amino Acids, Drugs and Explosives

Simone Mathias[1], Marius Amerio-Cox[1], Toni Jackson[1], David Douce[2], Bryan McCullough[2], Ashley Sage[2], Peter Luke[3], Carol Crean[1] and Patrick Sears[1*]

*Corresponding Author Email: p.sears@surrey.ac.uk

[1] School of Chemistry and Chemical Engineering, University of Surrey, Guildford GU2 7XH, UK [2] Waters Corporation, Stamford Avenue, Wilmslow SK9 4AX, UK [3] Mass Spec Analytical, Future Space UWE North Gate, Bristol BS34 8RB

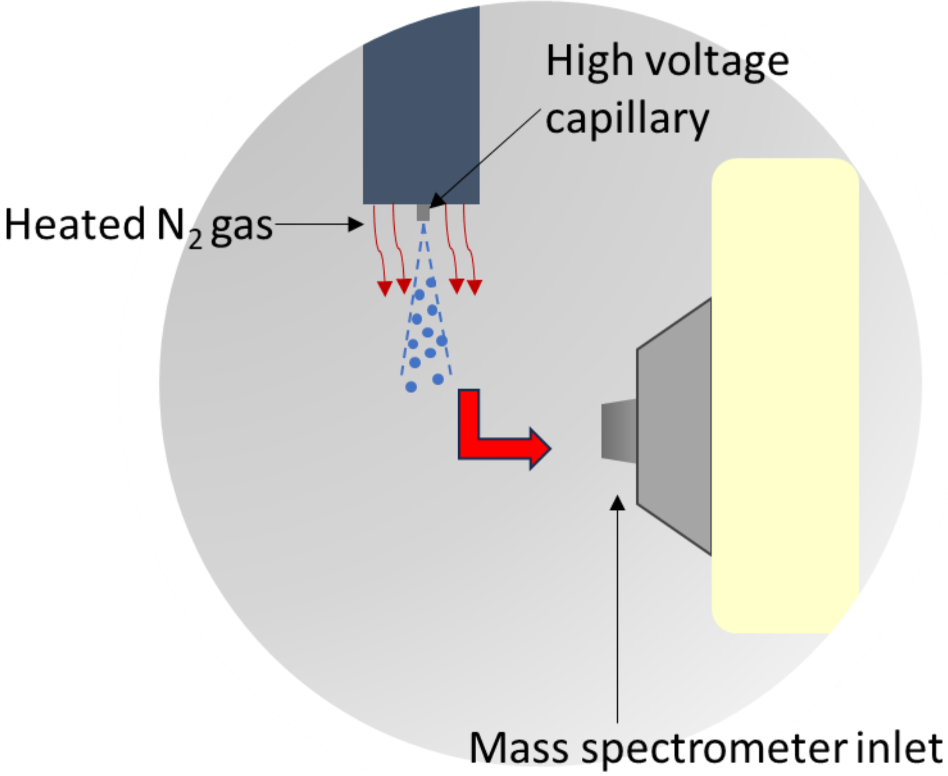


**Figure S1**: Schematic of the ESI source.

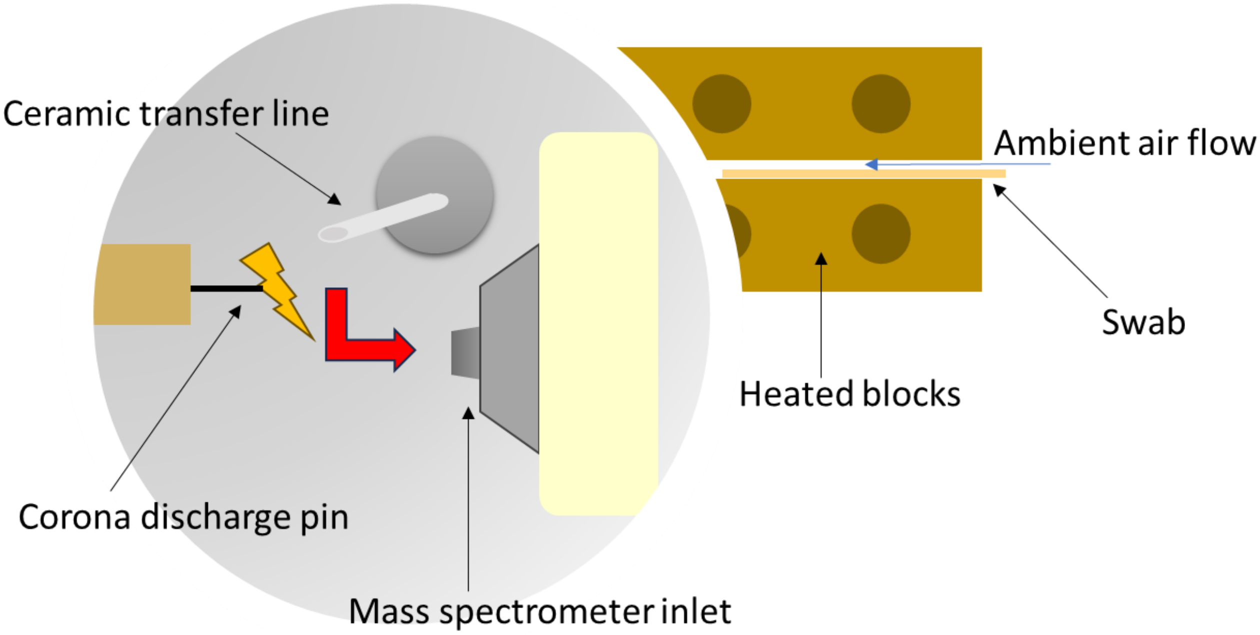


**Figure S2:** Schematic of the TDCD ionization source.

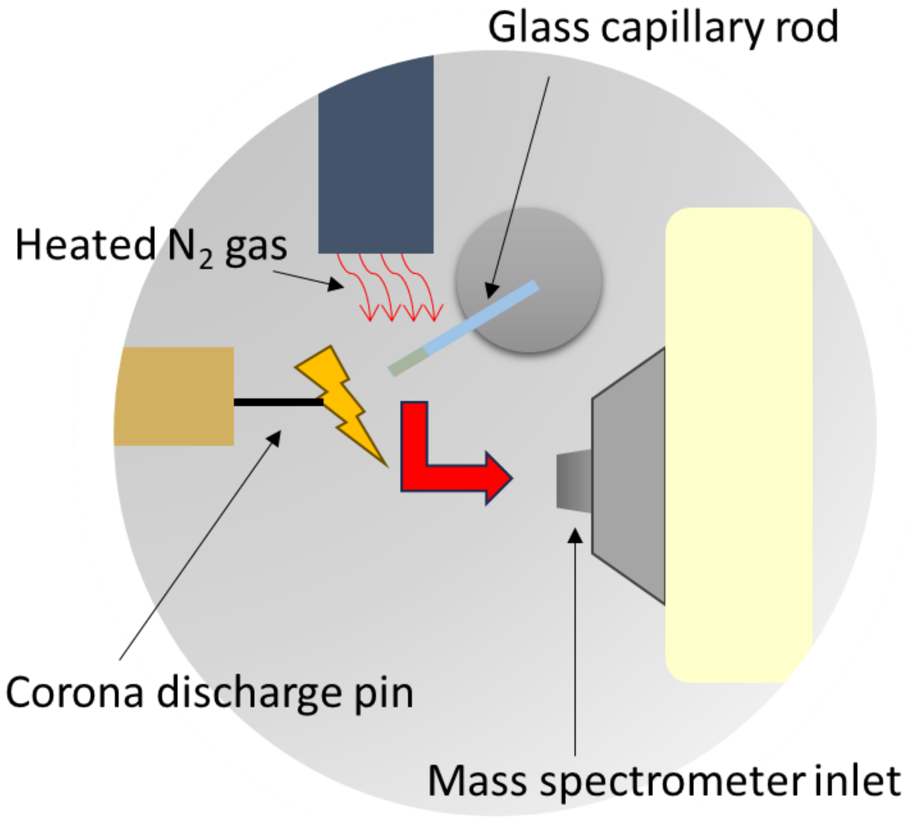


**Figure S3**: Schematic of the ASAP ionization source.

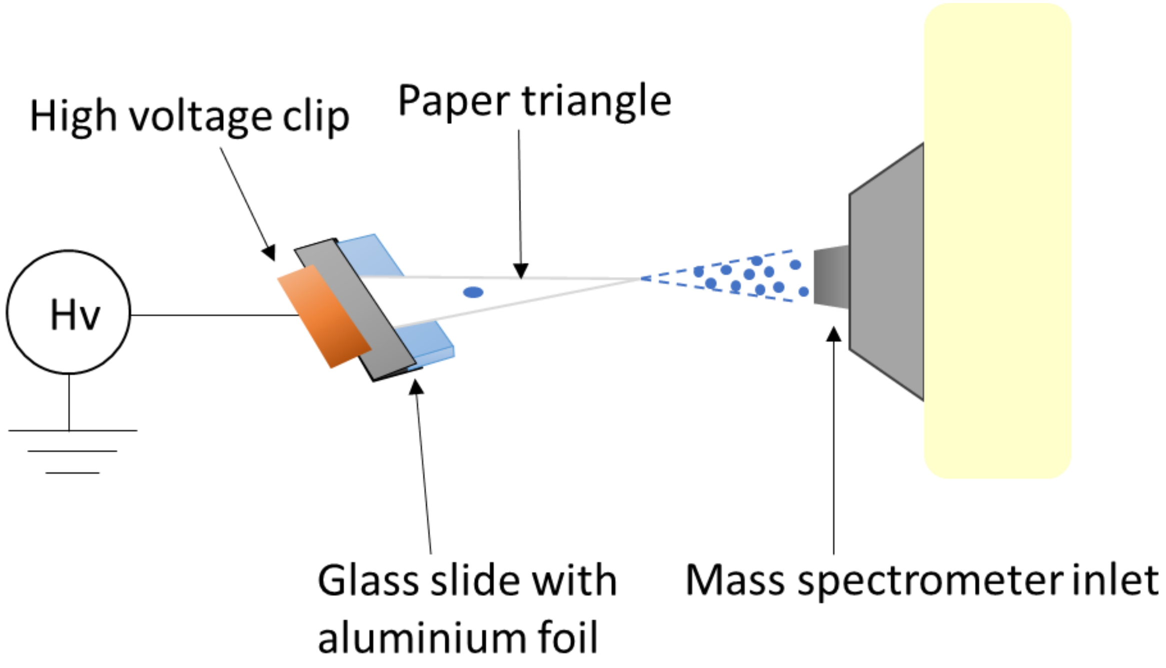


**Figure S4**: Schematic of the paper spray ionization source.

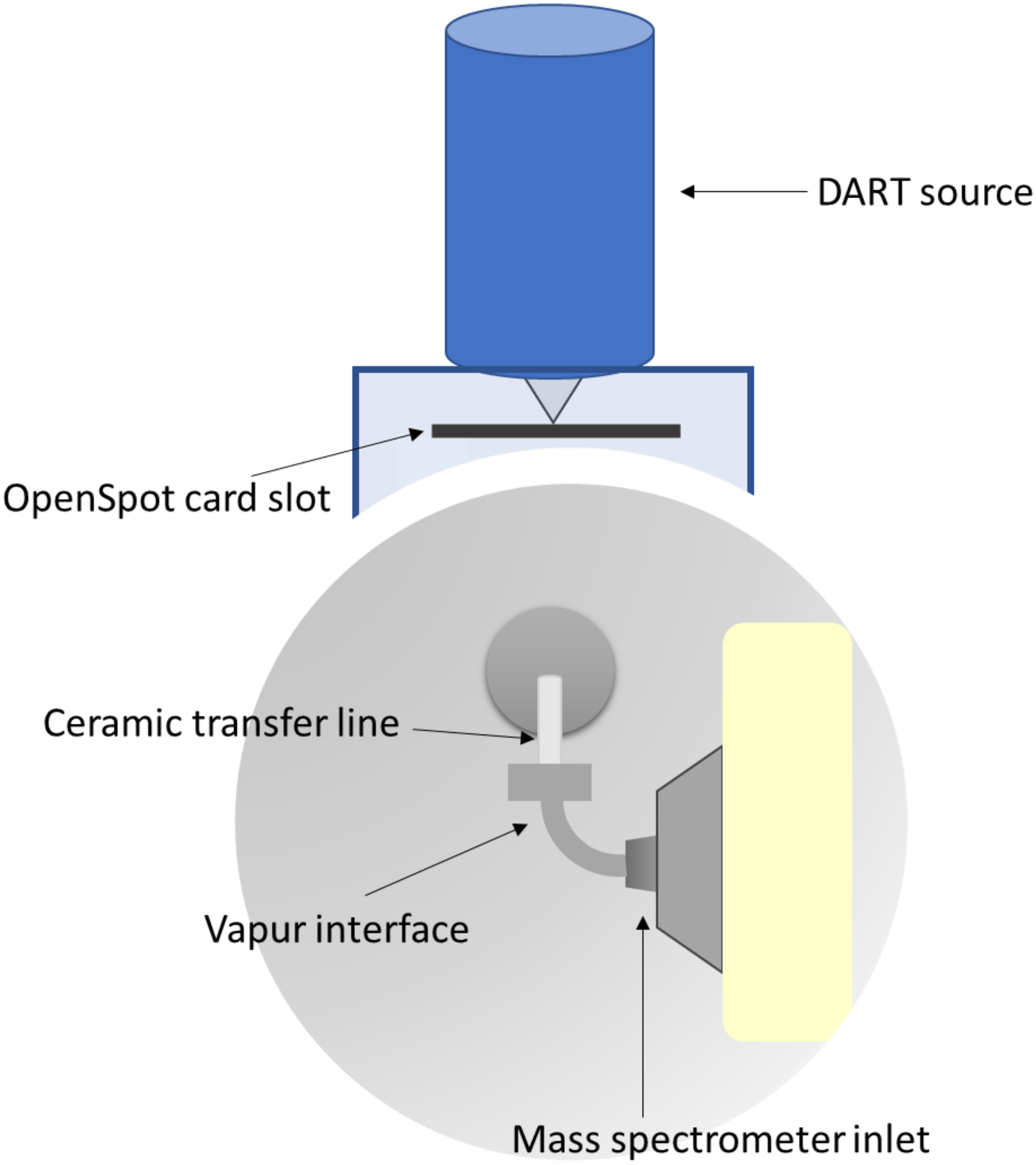


**Figure S5**: Schematic of the DART ionization source.

**Table S1**: Selected ion monitoring (SIM) methods with *m/z* value, assignment and associated cone voltage for each technique used.

| Analyte | *m/z* | Assignment | ASAP cone voltage (V) | TDCD cone voltage (V) | DART cone voltage (V) | Paper spray cone voltage (V) | ESI cone voltage (V) |
|---|---|---|---|---|---|---|---|
| **Amphetamine** | 136 | $[M+H]^+$ | 5 | 10 | 25 | 15 | 5 |
| | 119 | $[M-NH_2]^+$ | 5 | 20 | 50 | 40 | 15 |
| | 91 | $[M-NH_2-C_2H_3]^+$ | 5 | 25 | 80 | 20 | 25 |
| **Ketamine** | 240 | $[M(^{37}Cl)+H]^+$ | 15 | 12 | 20 | 10 | 5 |
| | 238 | $[M(^{35}Cl)+H]^+$ | 15 | 15 | 20 | 10 | 5 |
| | 220 | $[M-HO]^+$ | 35 | 30 | 55 | 35 | 30 |
| | 207 | $[M-CNH_4]^+$ | 25 | 30 | 50 | 30 | 25 |
| | 179 | $[M-CNH_4-CO]^+$ | 35 | 30 | 55 | 35 | 30 |
| | 125 | $[M-CNH_4-CO-C_4H_6]^+$ | 40 | - | 75 | 45 | - |
| **THC** | 315 | $[M+H]^+$ | 15 | 15 | 20 | 15 | 5 |
| | 259 | $[M-C_4H_7]^+$ | 35 | 40 | 60 | 40 | 35 |
| | 235 | $[M-C_6H_7]^+$ | 35 | 40 | 70 | 40 | - |
| | 193 | $[M-C_9H_{13}]^+$ | 35 | 40 | 45 | 40 | 40 |
| **Cocaine** | 304 | $[M+H]^+$ | 15 | 15 | 10 | 25 | 5 |
| | 182 | $[M-C_7H_5O_2]^+$ | 35 | 40 | 35 | 45 | 35 |
| | 105 | $[M-C_{10}H_{16}NO_3]^+$ | 45 | 50 | 55 | 55 | 55 |
| | 82 | $[M-C_{12}H_{13}O_4]^+$ | 40 | 50 | 55 | 55 | 55 |
| | 77 | $[M-C_{11}H_{16}NO_4]^+$ | 45 | 65 | 70 | 80 | 80 |
| **Phenylalanine** | 166 | $[M+H]^+$ | 10 | 5 | 35 | 20 | 5 |
| | 120 | $[M-CHO_2]^+$ | 20 | 20 | 70 | 25 | 25 |
| | 103 | $[M-CH_4NO_2]^+$ | 40 | 45 | 80 | 40 | 45 |
| **Leucine** | 132 | $[M+H]^+$ | 5 | 10 | 20 | 15 | 5 |
| | 86 | $[M-CH_2O_2]^+$ | 15 | 20 | 60 | 30 | 20 |
| **HMTD** | 229 | $[TMDDD+Na]^+$ | - | - | - | 15 | 10 |
| | 224 | $[TMDDD+NH_4]^+$ | - | - | 10 | 5 | - |
| | 213 | $[TMDDD+LI]^+$ | - | - | - | - | 15 |
| | 209 | $[M+H]^+$ | 5 | 5 | 5 | 15 | - |
| | 207 | $[TMDDD+H]^+$ | 10 | - | 20 | 20 | - |
| | 179 | $[TMDDD-CO]^+$ | 10 | 5 | 5 | 20 | - |
| | 145 | $[TMDDD-H_2O_2]^{+}$ - or $[M-CH_4O_3]^+$ | 10 | 10 | 10 | 20 | - |
| | 88 | $[TMDDD-C_3H_5NO_4]^+$ or $[M-C_3H_7NO_4]^+$ | - | 20 | 20 | - | - |
| **TNT** | 243 | $[M+O]^-$ | - | 5 | - | - | - |
| | 227 | $[M\bullet]^-$ | 5 | 5 | 10 | 20 | - |
| | 226 | $[M-H]^-$ | 20 | 15 | 5 | 10 | 15 |
| | 213 | $[M+O-NO]^-$ | 20 | - | 15 | - | - |
| | 210 | $[M-HO]^-$ | 15 | 15 | 20 | - | - |
| | 197 | $[M-NO]^-$ | 20 | 15 | 20 | 10 | - |

| Analyte | *m/z* | Assignment | ASAP cone voltage (V) | TDCD cone voltage (V) | DART cone voltage (V) | Paper spray cone voltage (V) | ESI cone voltage (V) |
|---|---|---|---|---|---|---|---|
| | 324 | $[M+C_2H_4N_3O_2]^-$ | 5 | - | 10 | - | - |
| | 284 | $[M+NO_3]^-$ | - | - | 5 | 5 | 5 |
| | 283 | $[M+NO_3-H]^-$ | 5 | - | - | - | - |
| | 268 | $[M+NO_2]^-$ | 5 | 5 | 5 | - | - |
| **RDX** | 267 | $[M+NO_2-H]^-$ | 5 | 5 | 5 | - | - |
| | 259 | $[M+^{37}Cl]^-$ | - | - | - | 5 | 5 |
| | 257 | $[M+^{35}Cl]^-$ | - | - | - | 5 | 5 |
| | 129 | $[M-HN_2O_4]^-$ | 5 | - | 20 | - | - |
| | 102 | $[M-CH_2N_3O_4]^-$ | 5 | - | 15 | - | - |
| | 349 | $[M+NO_3]^-$ | - | - | 5 | 5 | 5 |
| | 324 | $[M+^{37}Cl]^-$ | - | - | - | 5 | - |
| | 322 | $[M+^{35}Cl]^-$ | - | - | - | 5 | - |
| | 318 | $[M+CH_3OH-H]^-$ | - | - | - | - | 5 |
| | 304 | $[M-NO_2+HNO_3]^-$ | 10 | 5 | 15 | - | - |
| | 288 | $[M-NO_2+HNO_2]^-$ | - | - | - | - | 10 |
| **Tetryl** | 286 | $[M-H]^-$ | 5 | - | - | - | - |
| | 257 | $[M-NO]^-$ | 5 | 5 | 5 | - | - |
| | 241 | $[M-NO_2]^-$ | 5 | 5 | 5 | 10 | 10 |
| | 228 | $[M-NO_2-CH]^-$ | - | - | - | - | 10 |
| | 195 | $[M-NO_2-NO_2]^-$ | 20 | - | - | - | - |
| | 181 | $[M-NO_2-CH_2\ NO_2]^-$ | 20 | 15 | 20 | - | - |
| | 378 | $[M+NO_3]^-$ | 5 | 5 | 5 | 5 | 10 |
| | 376 | $[M+CO_3]^-$ | 5 | 5 | - | - | - |
| | 362 | $[M+NO_2]^-$ | 5 | 5 | 5 | - | - |
| **PETN** | 353 | $[M+^{37}Cl]^-$ | - | - | - | 5 | - |
| | 351 | $[M+^{35}Cl]^-$ | - | - | - | 5 | - |
| | 315 | $[M-H]^-$ | 5 | - | 5 | 5 | - |
| | 62 | $[NO_3]^-$ | - | - | 10 | - | - |

**Table S2**: Estimated LODs for paper spray for the target analytes.

| Analyte | LOD (pg) |
|---|---|
| Amphetamine | 100 |
| Ketamine | 300 |
| Cocaine | 80 |
| THC | 100 |
| Leucine | 300 |
| Phenylalanine | 200 |
| HMTD | 400 |
| PETN | 200 |
| Tetryl | 200 |
| TNT | 7000 |
| RDX | 100 |

**Table S3**: Masses at which an approximate linear relationship was established with the coefficient of determination ($R^2$) for each analyte using ASAP, TDCD or DART ionisation technique.

| Analyte | ASAP | | TDCD | | DART | | ESI | |
|---|---|---|---|---|---|---|---|---|
| | Mass (ng) | $R^2$ | Mass (ng) | $R^2$ | Mass (ng) | $R^2$ | Mass (ng) | $R^2$ |
| Amphetamine | 0.25 – 7.5 | 0.9015 | 5 - 100 | 0.9398 | 0.5 - 10 | 0.9843 | 0.008 - 12 | 0.9908 |
| Ketamine | 0.25 - 10 | 0.9904 | 1 - 100 | 0.9847 | 0.75 - 15 | 0.9864 | 0.001 - 14 | 0.9959 |
| Cocaine | 0.25 - 100 | 0.9968 | 0.25 - 100 | 0.9928 | 0.25 – 7.5 | 0.9267 | 0.001 - 14 | 0.9914 |
| THC | 0.25 - 100 | 0.9953 | 2 - 100 | 0.9940 | 0.5 - 20 | 0.9329 | 0.08 - 35 | 0.9979 |
| Leucine | 5 – 75 | 0.9450 | 2 - 75 | 0.9526 | 0.25 - 100 | 0.9840 | 0.06 – 31.5 | 0.9954 |
| Phenylalanine | 10 – 75 | 0.9135 | 10 – 75 | 0.9864 | 5 – 100 | 0.9852 | 0.01 - 35 | 0.9949 |
| HMTD | 0.5 - 50 | 0.9877 | 5 – 100 | 0.9925 | 1.5 - 100 | 0.9780 | 0.06 - 14 | 0.9912 |
| PETN | 1.5 - 75 | 0.9856 | 5 – 100 | 0.9949 | 0.25 - 25 | 0.9748 | 3.5 – 28 | 0.9861 |
| Tetryl | 0.25 - 25 | 0.9657 | 5 – 100 | 0.9921 | 0.25 – 25 | 0.9606 | 0.002 - 14 | 0.9914 |
| TNT | 0.25 – 5 | 0.9848 | 1 – 100 | 0.9956 | 0.25 – 5 | 0.9652 | 0.01 - 35 | 0.9929 |
| RDX | 0.5 - 100 | 0.9804 | 5 - 100 | 0.9856 | 1.5 - 100 | 0.9688 | 0.003 - 12 | 0.9841 |

**Table S4**: Precision (RSD) (n=5) for calibrator points covering the approximate linear range for ASAP, TDCD, DART and ESI.

| Analyte | ASAP | | TDCD | | DART | | ESI | |
|---|---|---|---|---|---|---|---|---|
| | Mass (ng) | RSD (%) | Mass (ng) | RSD (%) | Mass (ng) | RSD (%) | Mass (ng) | RSD (%) |
| Amphetamine | 0.25 | 21% | 5.0 | 5.4% | 0.5 | 44% | 0.008 | 1.6% |
| | 0.50 | 28% | 10.0 | 9.4% | 0.75 | 37% | 0.009 | 1.2% |
| | 0.75 | 14% | 15.0 | 19% | 1 | 35% | 0.01 | 2.6% |
| | 1.0 | 19% | 20.0 | 10% | 1.5 | 22% | 0.015 | 2.5% |
| | 1.5 | 21% | 25.0 | 11% | 2 | 16% | 0.02 | 2.3% |
| | 2.0 | 49% | 50.0 | 11% | 2.5 | 23% | 0.03 | 2.1% |
| | 2.5 | 25% | 75.0 | 9.3% | 3 | 14% | 0.04 | 1.7% |
| | 3.0 | 49% | 100.0 | 24% | 4 | 17% | 0.05 | 1.3% |
| | 4.0 | 41% | - | - | 5 | 30% | 0.06 | 1.8% |
| | 5.0 | 36% | - | - | 7.5 | 10% | 0.07 | 1.9% |
| | 7.5 | 38% | - | - | 10.0 | 13% | 0.08 | 0.5% |
| | - | - | - | - | - | - | 2.0 | 0.7% |
| | - | - | - | - | - | - | 4.0 | 0.6% |
| | - | - | - | - | - | - | 6.0 | 0.4% |
| | - | - | - | - | - | - | 8.0 | 0.7% |
| | - | - | - | - | - | - | 12.0 | 0.6% |
| Ketamine | 0.25 | 22% | 1 | 18% | 0.75 | 60% | 0.001 | 4.6% |
| | 0.50 | 33% | 1.5 | 26% | 1 | 19% | 0.002 | 3.1% |
| | 0.75 | 40% | 2 | 21% | 1.5 | 18% | 0.0025 | 2.5% |
| | 1.0 | 12% | 2.5 | 15% | 2 | 24% | 0.003 | 3.6% |
| | 1.5 | 19% | 3 | 16.% | 2.5 | 24% | 0.004 | 2.7% |
| | 2.0 | 26% | 4 | 9.3% | 3 | 19% | 0.005 | 2.1% |
| | 2.5 | 19% | 5 | 7.5% | 4 | 25% | 0.006 | 2.8% |
| | 3.0 | 18% | 7.5 | 6.1% | 5 | 17% | 0.007 | 1.4% |
| | 4.0 | 15% | 10 | 23% | 7.5 | 39% | 0.008 | 3.2% |
| | 5.0 | 30% | 15 | 12% | 10.0 | 26% | 0.009 | 1.8% |
| | 7.5 | 46% | 20 | 26% | 15 | 12% | 0.01 | 1.2% |
| | 10.0 | 29% | 25 | 11% | - | - | 2 | 0.4% |
| | - | - | 50 | 31% | - | - | 4 | 0.4% |
| | - | - | 75 | 14% | - | - | 6 | 0.3% |
| | - | - | 100 | 6.7% | - | - | 8 | 0.7% |
| | - | - | - | - | - | - | 10 | 0.6% |
| | - | - | - | - | - | - | 12 | 0.4% |
| | - | - | - | - | - | - | 14 | 0.7% |
| Cocaine | 0.25 | 20% | 0.25 | 7.1% | 0.25 | 53% | 0.001 | 6.6% |
| | 0.5 | 42% | 0.5 | 16% | 0.50 | 17% | 0.002 | 4.9% |
| | 0.75 | 38% | 0.75 | 20% | 0.75 | 34% | 0.0025 | 5.9% |
| | 1.0 | 22% | 1 | 9.3% | 1.0 | 34% | 0.003 | 3.8% |
| | 1.5 | 34% | 1.5 | 12% | 1.5 | 12% | 0.004 | 3.0% |
| | 2.0 | 62% | 2 | 17% | 2.0 | 46% | 0.005 | 2.3% |
| | 2.5 | 40% | 2.5 | 17% | 2.5 | 30% | 0.006 | 2.4% |
| | 3.0 | 35% | 3 | 15% | 3.0 | 27% | 0.007 | 2.6% |
| | 4.0 | 48% | 4 | 5.1% | 4.0 | 18% | 0.008 | 3.3% |
| | 5.0 | 14% | 5 | 21% | 5.0 | 12% | 0.009 | 2.4% |
| | 7.5 | 50% | 7.5 | 7.6% | 7.5 | 21% | 0.01 | 1.6% |
| | 10.0 | 37% | 10.0 | 35% | - | - | 2 | 0.9% |
| | 15.0 | 49% | 15 | 22% | - | - | 4 | 0.5% |
| | 20.0 | 26% | 20 | 14% | - | - | 6 | 0.5% |
| | 25.0 | 31% | 25 | 3.9% | - | - | 8 | 0.7% |
| | 50.0 | 35% | 50 | 26% | - | - | 10 | 0.6% |
| | 75.0 | 25% | 75 | 17% | - | - | 12 | 0.4% |
| | 100.0 | 22% | 100 | 18% | - | - | 14 | 0.4% |

| Analyte | ASAP | | TDCD | | DART | | ESI | |
|---|---|---|---|---|---|---|---|---|
| | Mass (ng) | RSD (%) | Mass (ng) | RSD (%) | Mass (ng) | RSD (%) | Mass (ng) | RSD (%) |
| THC | 0.25 | 37% | 2.0 | 11% | 0.5 | 41% | 0.08 | 3.4% |
| | 0.5 | 30% | 2.5 | 9.3% | 0.75 | 47% | 0.09 | 3.3% |
| | 0.75 | 30% | 3.0 | 10% | 1.0 | 13% | 0.1 | 2.3% |
| | 1 | 28% | 4.0 | 19% | 1.5 | 31% | 0.125 | 2.6% |
| | 1.5 | 22% | 5.0 | 61% | 2.0 | 39% | 0.15 | 2.1% |
| | 2 | 26% | 7.5 | 19% | 2.5 | 34% | 0.175 | 4.1% |
| | 2.5 | 42% | 10.0 | 14% | 3.0 | 40% | 0.2 | 3.2% |
| | 3 | 18% | 15.0 | 49% | 4.0 | 39% | 0.25 | 2.8% |
| | 4 | 17% | 20.0 | 16% | 5.0 | 32% | 0.3 | 1.9% |
| | 5 | 20% | 25.0 | 20% | 7.5 | 36% | 0.4 | 2.5% |
| | 7.5 | 35% | 50.0 | 16% | 10.0 | 30% | 0.5 | 3.3% |
| | 10.0 | 21% | 75.0 | 16% | 15.0 | 25% | 0.6 | 3.5% |
| | 15 | 19% | 100.0 | 27% | 20.0 | 27% | 0.7 | 1.8% |
| | 20 | 25% | - | - | - | - | 0.8 | 1.4% |
| | 25 | 20% | - | - | - | - | 3.5 | 3.7% |
| | 50 | 15% | - | - | - | - | 7 | 2.7% |
| | 75 | 17% | - | - | - | - | 10.5 | 1.8% |
| | 100 | 6.1% | - | - | - | - | 14 | 1.6% |
| | - | - | - | - | - | - | 17.5 | 1.4% |
| | - | - | - | - | - | - | 21 | 0.9% |
| | - | - | - | - | - | - | 24.5 | 0.9% |
| | - | - | - | - | - | - | 28 | 0.6% |
| | - | - | - | - | - | - | 31.5 | 1.7% |
| | - | - | - | - | - | - | 35 | 1.9% |
| Leucine | 5.0 | 36% | 2.0 | 20% | 0.25 | 55% | 0.06 | 1.6% |
| | 10.0 | 22% | 2.5 | 11% | 0.5 | 61% | 0.07 | 1.0% |
| | 15.0 | 23% | 3.0 | 17% | 0.75 | 40% | 0.08 | 2.2% |
| | 20.0 | 29% | 4.0 | 25% | 1.0 | 109% | 0.09 | 1.3% |
| | 25.0 | 46% | 5.0 | 21% | 1.5 | 37% | 0.1 | 2.6% |
| | 50.0 | 75% | 7.5 | 10% | 2.0 | 50% | 0.125 | 2.1% |
| | 75.0 | 40% | 10.0 | 10% | 2.5 | 71% | 0.15 | 3.6% |
| | - | - | 15.0 | 18% | 3.0 | 75% | 0.175 | 2.5% |
| | - | - | 20.0 | 34% | 4.0 | 35% | 0.2 | 2.5% |
| | - | - | 25.0 | 18% | 5.0 | 50% | 0.25 | 1.9% |
| | - | - | 50.0 | 51% | 7.5 | 15% | 0.3 | 1.4% |
| | - | - | 75.0 | 8.3% | 10.0 | 23% | 0.35 | 1.9% |
| | - | - | - | - | 15.0 | 28% | 3.5 | 3.8% |
| | - | - | - | - | 20.0 | 15% | 7 | 2.7% |
| | - | - | - | - | 25.0 | 4.6% | 10.5 | 0.9% |
| | - | - | - | - | 50.0 | 11% | 14 | 1.8% |
| | - | - | - | - | 75.0 | 12% | 17.5 | 1.0% |
| | - | - | - | - | 100.0 | 8.6% | 21 | 0.8% |
| | - | - | - | - | - | - | 24.5 | 0.9% |
| | - | - | - | - | - | - | 28 | 1.1% |
| | - | - | - | - | - | - | 31.5 | 0.4% |

| Analyte | ASAP | | TDCD | | DART | | ESI | |
|---|---|---|---|---|---|---|---|---|
| | Mass (ng) | RSD (%) | Mass (ng) | RSD (%) | Mass (ng) | RSD (%) | Mass (ng) | RSD (%) |
| Phenylalanine | 10.0 | 60% | 10.0 | 10% | 5.0 | 38% | 0.01 | 2.6% |
| | 15.0 | 56% | 15.0 | 7.9% | 10.0 | 49% | 0.02 | 1.9% |
| | 20.0 | 36% | 20.0 | 10% | 15.0 | 27% | 0.03 | 0.8% |
| | 25.0 | 45% | 25.0 | 5.3% | 20.0 | 18% | 0.04 | 2.0% |
| | 50.0 | 73% | 50.0 | 14% | 25.0 | 4.7% | 0.045 | 0.8% |
| | 75.0 | 67% | 75.0 | 11% | 50.0 | 16% | 0.05 | 1.3% |
| | - | - | - | - | 75.0 | 14% | 0.06 | 1.0% |
| | - | - | - | - | 100.0 | 16% | 0.07 | 1.1% |
| | - | - | - | - | - | - | 0.08 | 1.7% |
| | - | - | - | - | - | - | 0.09 | 1.0% |
| | - | - | - | - | - | - | 0.1 | 0.9% |
| | - | - | - | - | - | - | 0.2 | 2.5% |
| | - | - | - | - | - | - | 0.3 | 1.9% |
| | - | - | - | - | - | - | 3.5 | 0.5% |
| | - | - | - | - | - | - | 7 | 1.9% |
| | - | - | - | - | - | - | 10.5 | 0.3% |
| | - | - | - | - | - | - | 14 | 0.3% |
| | - | - | - | - | - | - | 17.5 | 0.3% |
| | - | - | - | - | - | - | 21 | 0.3% |
| | - | - | - | - | - | - | 24.5 | 1.8% |
| | - | - | - | - | - | - | 28 | 0.7% |
| | - | - | - | - | - | - | 31.5 | 0.5% |
| | - | - | - | - | - | - | 35 | 0.1% |
| HMTD | 0.5 | 27% | 5.0 | 9.0% | 1.5 | 42% | 0.06 | 5.2% |
| | 0.75 | 38% | 10.0 | 4.9% | 2.0 | 35% | 0.07 | 2.5% |
| | 1 | 63% | 15.0 | 5.2% | 2.5 | 14% | 0.08 | 3.6% |
| | 1.5 | 36% | 20.0 | 5.0% | 3.0 | 29% | 0.09 | 4.6% |
| | 2 | 22% | 25.0 | 4.9% | 4.0 | 12% | 0.1 | 3.4% |
| | 2.5 | 21% | 50.0 | 5.5% | 5.0 | 29% | 0.125 | 4.8% |
| | 3 | 28% | 75.0 | 9.4% | 7.5 | 20% | 0.15 | 3.2% |
| | 4 | 53% | 100.0 | 7.0% | 10.0 | 16% | 0.175 | 4.2% |
| | 5 | 49% | - | - | 15.0 | 41% | 0.2 | 1.7% |
| | 7.5 | 43% | - | - | 20.0 | 13% | 0.25 | 3.6% |
| | 10.0 | 46% | - | - | 25.0 | 16% | 0.3 | 3.4% |
| | 15 | 29% | - | - | 50.0 | 13% | 0.35 | 2.1% |
| | 20 | 25% | - | - | 75.0 | 19% | 2 | 2.6% |
| | 25 | 23% | - | - | 100.0 | 8.9% | 4 | 1.1% |
| | 50 | 41% | - | - | - | - | 6 | 2.5% |
| | - | - | - | - | - | - | 8 | 2.1% |
| | - | - | - | - | - | - | 10 | 1.8% |
| | - | - | - | - | - | - | 12 | 1.3% |
| | - | - | - | - | - | - | 14 | 1.5% |
| PETN | 1.5 | 56% | 5.0 | 18% | 0.25 | 58% | 3.5 | 12% |
| | 2 | 39% | 10.0 | 5.2% | 0.5 | 127% | 7.0 | 12% |
| | 2.5 | 49% | 15.0 | 6.3% | 0.75 | 104% | 10.5 | 11% |
| | 3 | 52% | 20.0 | 10% | 1.0 | 67% | 14.0 | 10% |
| | 4 | 81% | 25.0 | 10% | 1.5 | 34% | 17.5 | 10% |
| | 5 | 76% | 50.0 | 14% | 2.0 | 103% | 21.0 | 9.0% |
| | 7.5 | 44% | 75.0 | 21% | 2.5 | 98% | 24.5 | 10% |
| | 10.0 | 84% | 100.0 | 14% | 3.0 | 53% | 28.0 | 13% |
| | 15 | 100% | - | - | 4.0 | 29% | - | - |
| | 20 | 76% | - | - | 5.0 | 94% | - | - |
| | 25 | 45% | - | - | 7.5 | 110% | - | - |
| | 50 | 24% | - | - | 10.0 | 64% | - | - |
| | 75 | 55% | - | - | 15.0 | 27% | - | - |
| | - | - | - | - | 20.0 | 45% | - | - |
| | - | - | - | - | 25.0 | 28% | - | - |

| Analyte | ASAP | | TDCD | | DART | | ESI | |
|---|---|---|---|---|---|---|---|---|
| | Mass (ng) | RSD (%) | Mass (ng) | RSD (%) | Mass (ng) | RSD (%) | Mass (ng) | RSD (%) |
| Tetryl | 0.25 | 36% | 5.0 | 5.6% | 0.25 | 86% | 0.002 | 18% |
| | 0.5 | 28% | 10.0 | 1.0% | 0.5 | 39% | 0.003 | 14% |
| | 0.75 | 38% | 15.0 | 6.7% | 0.75 | 13% | 0.004 | 16% |
| | 1.0 | 39% | 20.0 | 3.4% | 1.0 | 50% | 0.005 | 14% |
| | 1.5 | 25% | 25.0 | 6.9% | 1.5 | 36% | 0.006 | 8.1% |
| | 2.0 | 26% | 50.0 | 12% | 2.0 | 44% | 0.007 | 22% |
| | 2.5 | 39% | 75.0 | 9.1% | 2.5 | 47% | 0.008 | 7.8% |
| | 3.0 | 25% | 100.0 | 4.7% | 3.0 | 40% | 0.009 | 18% |
| | 4.0 | 36% | - | - | 4.0 | 43% | 0.01 | 9.4% |
| | 5.0 | 11% | - | - | 5.0 | 30% | 2 | 1.7% |
| | 7.5 | 22% | - | - | 7.5 | 23% | 4 | 0.7% |
| | 10.0 | 18% | - | - | 10.0 | 32% | 6 | 1.1% |
| | 15.0 | 25% | - | - | 15.0 | 18% | 8 | 0.3% |
| | 20.0 | 12% | - | - | 20.0 | 23% | 10 | 1.0% |
| | 25.0 | 22% | - | - | 25.0 | 32% | 12 | 1.2% |
| | - | - | - | - | - | - | 14 | 1.7% |
| TNT | 0.25 | 23% | 1 | 14% | 0.25 | 42% | 0.01 | 20% |
| | 0.5 | 43% | 1.5 | 2.8% | 0.5 | 20% | 0.02 | 8.7% |
| | 0.75 | 26% | 2 | 14% | 0.75 | 56% | 0.03 | 14% |
| | 1.0 | 24% | 2.5 | 7.4% | 1.0 | 54% | 0.04 | 8.2% |
| | 1.5 | 22% | 3 | 10% | 1.5 | 38% | 0.05 | 6.8% |
| | 2.0 | 16% | 4 | 17% | 2.0 | 45% | 0.06 | 12% |
| | 2.5 | 14% | 5 | 15% | 2.5 | 49% | 0.07 | 5.3% |
| | 3.0 | 26% | 7.5 | 18% | 3.0 | 33% | 0.08 | 5.5% |
| | 4.0 | 18% | 10 | 11% | 4.0 | 24% | 0.09 | 2.2% |
| | 5.0 | 14% | 15 | 12% | 5.0 | 21% | 0.1 | 2.4% |
| | - | - | 20 | 3.8% | - | - | 3.5 | 1.4% |
| | - | - | 25 | 12% | - | - | 7 | 0.9% |
| | - | - | 50 | 5.7% | - | - | 10.5 | 1.8% |
| | - | - | 75 | 12% | - | - | 14 | 2.1% |
| | - | - | 100 | 5.1% | - | - | 17.5 | 1.8% |
| | - | - | - | - | - | - | 21 | 1.8% |
| | - | - | - | - | - | - | 24.5 | 0.8% |
| | - | - | - | - | - | - | 28 | 1.2% |
| | - | - | - | - | - | - | 31.5 | 1.7% |
| RDX | 0.5 | 58% | 5.0 | 20% | 1.5 | 68% | 0.003 | 5.0% |
| | 0.75 | 55% | 10.0 | 15% | 2.0 | 91% | 0.004 | 3.2% |
| | 1.0 | 48% | 15.0 | 13% | 2.5 | 16% | 0.005 | 3.1% |
| | 1.5 | 85% | 20.0 | 9.1% | 3.0 | 61% | 0.006 | 6.2% |
| | 2.0 | 78% | 25.0 | 13% | 4.0 | 48% | 0.007 | 3.6% |
| | 2.5 | 80% | 50.0 | 7.7% | 5.0 | 44% | 0.008 | 4.9% |
| | 3.0 | 34% | 75.0 | 11% | 7.5 | 39% | 0.009 | 3.9% |
| | 4.0 | 43% | 100.0 | 14% | 10.0 | 35% | 0.01 | 3.3% |
| | 5.0 | 82% | - | - | 15.0 | 41% | 2 | 1.1% |
| | 7.5 | 58% | - | - | 20.0 | 7.1% | 4 | 0.5% |
| | 10.0 | 13% | - | - | 25.0 | 36% | 6 | 2.3% |
| | 15.0 | 43% | - | - | 50.0 | 24% | 8 | 0.8% |
| | 20.0 | 42% | - | - | 75.0 | 22% | 10 | 1.0% |
| | 25.0 | 7.4% | - | - | 100.0 | 21% | 12 | 0.6% |
| | 50.0 | 45% | - | - | - | - | - | - |
| | 75.0 | 43% | - | - | - | - | - | - |
| | 100.0 | 32% | - | - | - | - | - | - |

## Equations and Calculations

### Calculating the LOD

$$LOD = X_{b1} + 3S_{b1}$$

Where $X_{b1}$ is the mean blank response and $S_{b1}$ is the standard deviation of the blank. Analyte is considered detected when the response is above the value given by the above equation.

The analyte response of the lowest mass detected was divided by the calculated LOD response to give an estimated signal-to-noise. The lowest mass detected was then divided by the estimated signal-to-noise to produce an estimated LOD in ng.

### Relative Standard Deviation

$$RSD\ \% = \frac{S}{\bar{x}} \times 100$$

Where S is the standard deviation and $\bar{x}$ is the mean.
